# The Contribution of Emotional Partners to Sexual Risk Taking and Violence among Female Sex Workers in Mombasa, Kenya: A Cohort Study

**DOI:** 10.1371/journal.pone.0068855

**Published:** 2013-08-07

**Authors:** Stanley Luchters, Marlise L. Richter, Wilkister Bosire, Gill Nelson, Nzioki Kingola, Xu-Dong Zhang, Marleen Temmerman, Matthew F. Chersich

**Affiliations:** 1 Centre for International Health, Burnet Institute, Melbourne, Australia; 2 International Centre for Reproductive Health (ICRH), Department of Obstetrics and Gynaecology, Ghent University, Ghent, Belgium; 3 School of Public Health, Faculty of Health Sciences, University of the Witwatersrand, Johannesburg, South Africa; 4 Department of Epidemiology and Preventive Medicine, School of Public Health and Preventive Medicine, Faculty of Medicine, Nursing and Health Sciences, Monash University, Melbourne, Australia; 5 African Centre for Migration & Society, University of the Witwatersrand, Johannesburg, South Africa; 6 International Centre for Reproductive Health (ICRH), Mombasa, Kenya; 7 Centre for Health Policy, School of Public Health, Faculty of Health Sciences, University of the Witwatersrand, Johannesburg, South Africa; Catholic University of Sacred Heart of Rome, Italy

## Abstract

**Objectives:**

To assess sexual risk-taking of female sex workers (FSWs) with emotional partners (boyfriends and husbands), compared to regular and casual clients. Experiences of violence and the degree of relationship control that FSWs have with emotional partners are also described.

**Design:**

Cohort study with quarterly follow-up visit over 12-months.

**Methods:**

Four hundred HIV-uninfected FSWs older than 16 years were recruited from their homes and guesthouses in Mombasa, Kenya. A structured questionnaire assessed participant characteristics and study outcomes at each visit, and women received risk-reduction counselling, male and female condoms, and HIV testing.

**Results:**

Four or more unprotected sex acts in the past week were reported by 21.3% of women during sex with emotional partners, compared to 5.8% with regular and 4.8% with casual clients (*P*<0.001). Total number of unprotected sex acts per week was 5–6-fold higher with emotional partners (603 acts with 259 partners) than with regular or casual clients (125 acts with 456, and 98 acts with 632 clients, respectively; *P*<0.001). Mostly, perceptions of “trust” underscored unprotected sex with emotional partners. Low control over these relationships, common to many women (36.9%), was linked with higher partner numbers, inconsistent condom use, and being physically forced to have sex by their emotional partners. Half experienced sexual or physical violence in the past year, similarly associated with partner numbers and inconsistent condom use.

**Conclusions:**

High-risk sexual behaviour, low control and frequent violence in relationships with emotional partners heighten FSWs' vulnerability and high HIV risk, requiring targeted interventions that also encompass emotional partners.

## Introduction

In sub-Saharan Africa an estimated 0.7% to 4.3% of women exchange sex for money, goods or favours [1]. In low-income and middle-income countries in particular, these women carry a disproportionate burden of HIV and other sexually transmitted infections (STIs), with HIV risk about 12-fold higher than women in the general population [2]. Though the exact extent of the contribution is contested, sex workers, their clients and emotional partners play an important role in HIV transmission in sub-Saharan Africa [3], [4]. Importantly, studies have also shown that it is possible to have multiple sexual partners, and not contract HIV or other STIs, if condoms are used consistently [5]. Thus, the factors that enable or hinder unprotected sex with commercial and emotional partners are key to understanding the HIV risk that sex workers face.

HIV prevention initiatives in sex work settings overwhelming focus on sex workers, with few efforts to target sex worker clients, and virtually none addressing their emotional partners [6]. This is mostly because sex workers are easier to locate than their sexual partners, but reinforces notions of sex workers as disease vectors. Further, it locates responsibility for condom use in this group, who may not be the main decision-makers in their sexual relations, and may have low control over partner behaviour. Emotional partners of female sex workers (FSWs) are of particular concern due to their high levels of sexual risk behaviour [7]–[9] and they may contribute considerably to HIV transmission in the context of sex work [10]. Additional evidence for the role played by emotional partners of FSWs, and the often complex interaction between FSWs and these partners may stimulate research and programmatic interventions in this area.

Few studies have directly examined the role of emotional partners in FSWs' HIV risk [11]–[13]. This prospective cohort study among women with high-risk behaviour for HIV infection was conducted to estimate the annual HIV incidence and evaluate the feasibility of establishing a new site for microbicide clinical trials in Mombasa, Kenya. The analysis presented here compared sexual behaviour of FSWs with different partner types within this cohort, specifically emotional partners, and regular and casual clients. In addition, we assess the level of control that FSWs have in their relationships with emotional partners, and describe experiences of violence within these relationships.

## Methods

### Study setting and population

The study enrolled FSWs in Mombasa, a major economic centre in Kenya and East Africa, with busy port, rail and industrial enterprises, and also host to tourists from around the world. A capture-recapture enumeration of FSWs in 2010 estimated that there were over 18,000 FSWs in Mombasa (unpublished data). Convenience sampling was used, with FSWs recruited from their homes and guesthouses in two divisions of the city, which were divided into 11 zones; each was allocated a field worker. Field workers were familiar with their respective areas and responsible for inviting women to enrol in the study and maintain contact with them throughout the study period. The research team conducted study assessments from two research locations in Chaani (primary health centre) and Kisauni division (FSW drop-in centre). To be eligible, FSWs had to be HIV-uninfected, aged 16 years or older, not currently pregnant as assessed by self-report and laboratory screening, able and willing to provide written informed consent for study participation, and provide adequate locator information for tracing. Those planning to travel or relocate from the study areas, or participating in other HIV intervention studies, were excluded from participation.

### Data collection and assessments

Eligible women were followed over 12 months, with quarterly study visits. The sample size and follow-up duration were selected for the purposes of quantifying the HIV incidence in this population, and thus to inform sample size estimations for future HIV prevention trials in this population. A structured questionnaire was administered at each visit by a trained research assistant to collect data on socio-demographics (baseline), sexual behaviour with different types of sexual partners (quarterly), and relationship information (baseline and endline). Local staff translated the English questionnaires into Swahili, which were field tested before use. Questionnaires were held in English or Swahili.

HIV and pregnancy testing was done at each visit. HIV status was determined by using two negative HIV rapid tests performed in parallel with Uni-Gold™ HIV (Trinity Biotech plc, Bray, Ireland) and Determine™ HIV-1/2 (Abbott Laboratories by Abbott Japan Co Ltd, Minato-Ku, Tokyo, Japan). If the result of these were discordant, an HIV ELISA was performed at a laboratory at Coast Provincial General Hospital for confirmation. For participants sero-converting during the study, a polymerase chain reaction (PCR) HIV test was done on the last antibody negative blood sample to improve estimation of the timing of infection. HIV-infected women were referred for free antiretroviral treatment and care services.

Gynaecological examination and STI screening by trained clinicians were done at baseline, after 12 months, and at other visits if clinically indicated. Syphilis infection was detected with a rapid plasma reagin test (Human GmbH, Wiesbaden, Germany). Infection with *Trichomonas vaginalis* was determined by wet mount.

Participants received STI treatment according to local guidelines or were referred to health services when needed. Voluntary HIV testing and counselling, and contraceptive and risk-reduction counselling were provided. Contraceptives, including male and female condoms, were offered free of charge. The Kenyatta National Hospital Ethics and Research Committee approved the study protocol (P199/11/2005). All participants provided written informed consent. External monitoring was conducted, verifying available source documentation with study-specific clinical record forms.

### Study measures

A FSW was defined as a woman reporting to having had sexual intercourse at least once in the past three months, and having received money in exchange for it as part of her livelihood in the last six months. The questionnaire categorized each of the sexual partners of FSWs as a casual client (an occasional client or stranger who pays to have sex), a regular client (someone with whom the woman does not have an emotional relation, but who does not necessarily have to pay for sex each time), or an emotional partner (a boyfriend or husband, with whom the woman has an emotional attachment and who does not have to pay for sex every time).

For each partner type, women reported the total number of sex acts in the past week and condom use at each act. Condom use in the past three months was classified as inconsistent when women reported “never”, “sometimes”, or only using condoms “most of the time”. We also report the proportion of women who never used condoms, a subset of the inconsistent category. Other measures of sexual behaviour were: reasons for condom use, age-discordance with partner; and being physically forced to have sex by a boyfriend or husband.

We administered an adaptation [14] of the Relationship Control Subscale from the Sexual Relationship Power Scale [15]. The 12-item questionnaire assessed women's subjective experiences of being controlled by an emotional partner. Participants responded to each item on a four-point Likert scale, ranging from 1 (strongly agree) to 4 (strongly disagree). Total cumulative scores ranged from 12 (lowest perceived relationship control) to 48 (highest perceived relationship control). Scores were categorized as low relationship control (score 12–24), medium control (score 25–36) and high control (37–48). Experience of violence from any partner was assessed through a 19-item questionnaire, used by Dunkle et al [14], [16], which drew on the WHO violence against women instrument [17]. Information was collected on six different types of sexual, physical and other forms of violence, whether this had occurred in the past 12 months and, if so, how often (once, few, many times). Additional details about the perpetrator were obtained when women reported being physically forced to have sex when she didn't want to.

### Data management and analysis

Data were double entered by separate clerks and analysed using Stata SE 11.0 (Stata Corporation, College Station, TX, USA). Descriptive analysis of population characteristics assessed the distribution of continuous variables and the frequency distribution of categorical variables in contingency tables. For each partner type, generalized estimating equations were used to assess changes in condom use over the five study visits.

Sexual behaviour outcomes were analysed both at the FSW level and for all the partners she reported (partner-level analysis for each partner type). Sexual behaviour outcomes with emotional partners were compared with regular and casual clients, using a Poisson regression analysis for discrete count variables or ordinal regression analysis for ordered categorical variables, and controlled for multiple measures on the same subject. To assess associations between the three levels of relationship control and behavioural outcomes, Pearson Chi-square tests for trend were used for binary outcomes, and Kruskal Wallis tests for non-normal distribution of continuous variables.

## Results

In total, 400 women enrolled over a four-month accrual period and were followed-up for 12 months from May 2006 to September 2007. The mean age of participants was 25.1 years (SD = 5.2) and a third of women (34.8%, table 1) followed the Muslim religion. There was little evidence of migration or mobility as nearly all women were Kenyan (97.5%), and three quarters (76.3%) of participants had stayed at the same residence for the past two years. Only 11 women (2.8%) were currently married or cohabiting, 72.0% (288/400) were single, and 25.3% were divorced, separated or widowed (101/400). Having one or more children was reported by about 80% of women, and nearly three quarters of respondents (71.6%; 285/398) had an emotional partner at study enrolment. Of enrolled participants, 91.8% were retained in the study over 12 months (367/400).

**Table 1:** Characteristics of 400 FSWs at enrolment in the prospective cohort study in Mombasa, Kenya (2006–2007).

Variable	Sub-category	% (n/N)*
**Age**, mean years (sd)		25.1 (5.2)
**Nationality**	Kenyan	97.5% (390/400)
	Tanzanian/Ugandan	2.5% (10/400)
**Mobility in last 2 years**	Never changed residence	76.3% (302/396)
	Changed residence once	13.6% (54/396)
	Changed residence twice or more times	10.1% (40/396)
**Religious affiliation**	Catholic	25.5% (102/400)
	Protestant/other‡	39.8% (159/400)
	Muslim	34.8% (139/400)
**Highest education level**	None or primary incomplete	42.3% (169/400)
	Primary school	27.8% (111/400)
	Secondary or tertiary level	30.0% (120/400)
**Marital status**	Single	72.0% (288/400)
	Married or cohabiting	2.8% (11/400)
	Separated, divorced or widowed	25.3% (101/400)
**Currently has emotional partner**		71.6% (285/398)
**Duration of sex work**, median years (IQR) n = 398		4 (2–7)
**Part-time sex worker**		49.8% (199/400)
**Weekly income from sex work**	≤500 Kenya Shillings∧	23.9% (95/397)
	501–1000	30.2% (120/397)
	1001–2000	26.2% (104/397)
	>2000	19.7% (78/397)
**Number of live children**	0	19.8% (79/400)
	1	38.0% (152/400)
	2–3	34.0% (136/400)
	≥4	8.3% (33/400)
**Substances use (ever use - multiple response)**	Alcohol	72.3% (289/400)
	Cannabis	12.8% (51/400)
	Khat	35.8% (143/400)
**Perceived risk of acquiring HIV**	No risk	22.4% (89/398)
	Small risk	19.6% (78/398)
	Moderate risk	12.3% (49/398)
	Great risk	29.4% (117/398)
	Don't know	16.3% (65/398)

IQR inter-quartile range. sd standard deviation.

* % (n/N) unless otherwise stated.

‡ Five participants indicated ‘other’ religion.

∧ Exchange rate of 500 Kenya Shillings = 4.12 Euro.

### HIV/STI incidence and sexual behaviour according to partner type

**Figure 1:**
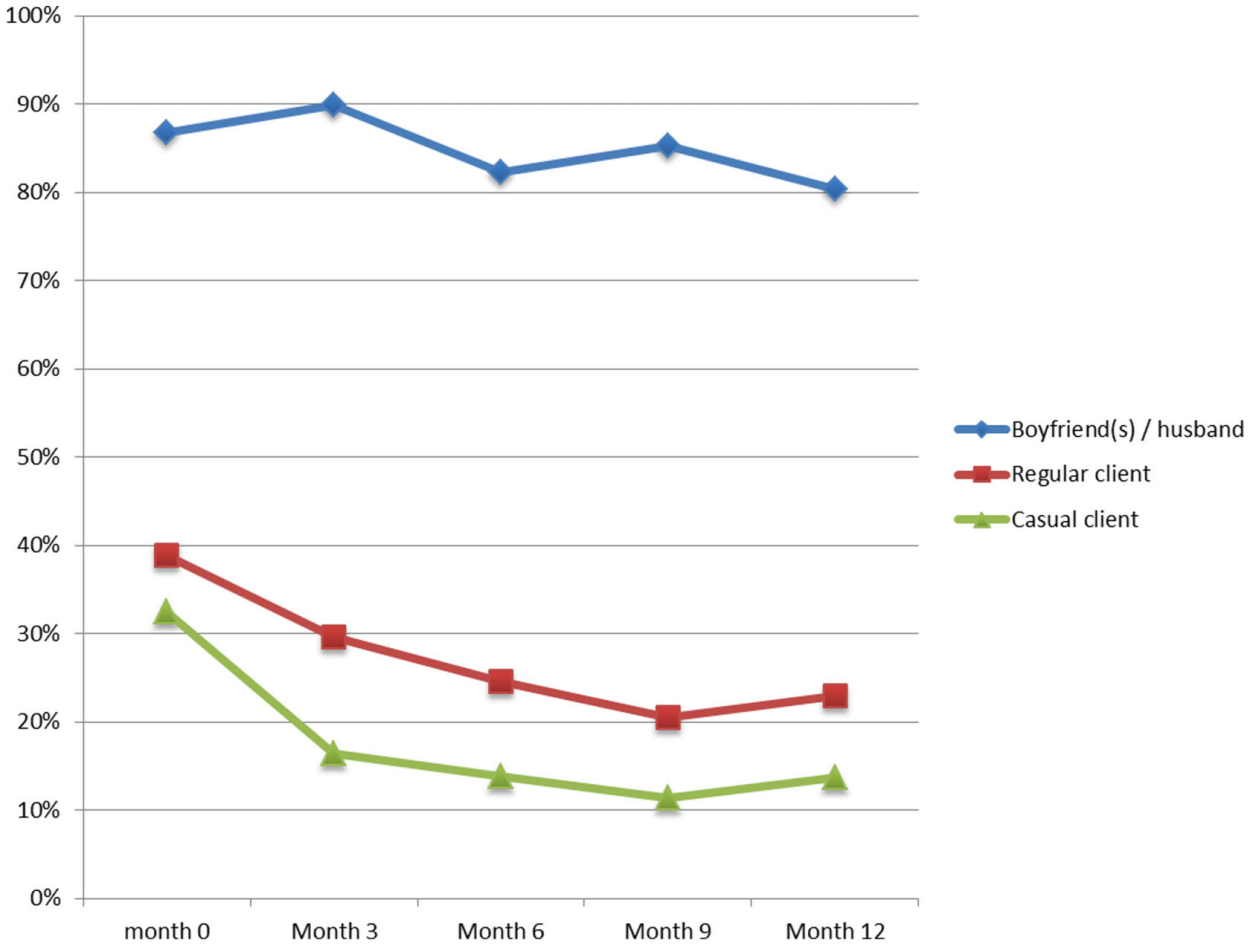
FSWs reporting inconsistent condom use in past 3 months. Percentage of FSWs who reported inconsistent condom use in the past 3 months with emotional partners, regular clients and casual clients at 3-monthly visits between May 2006 and September 2007.

At study enrolment, 62.9% (180/286) of women reported never using a condom during sex with their emotional partners, while only 5.8% (23/394) and 3.6% (13/360) of women had never used a condom with their regular clients and casual clients, respectively (data not shown). Over the 12-month study period, the proportion of sex workers reporting inconsistent condom use (three month recall period) with emotional partners decreased from nearly 90% to about 80% (*P* = 0.007, figure 1, table S1). Even more marked reductions in inconsistent condom use were seen with regular clients (from 39% at baseline to 23% after 12 months; *P*<0.001) and with casual partners (from 33% at baseline to 14% after 12 months; *P*<0.001; figure 1, table S1). After 12 months follow-up, respondents had had sex with a median of one emotional partner, two regular clients and two casual partners in the preceding week (table 2). Four or more unprotected sex acts in the past week was mentioned by 21.3% (51/239) of women during sex with emotional partners, while this was cited by 5.8% (12/208) and 4.8% (11/228) of women for sex with regular and casual clients, respectively (*P*<0.001; data not shown).

**Table 2:** Number of partners and sex acts, condom use and age discordance between FSWs and their emotional partners and clients after a 12-month cohort study (2006–2007).

Variable	Sub-category	Boyfriend(s) or husband A (n = 239)	Regular clients B(n = 208)	Casual clients C (n = 228)	*P value* A vs B	*P value* A vs C
Female sex worker level analysis						
**Number of partners in past week**, median (IQR)		1 (1-1, 1–4)	2 (1–3, 1–7)	2 (2–3, 1–17)	<0.001^a^	<0.001^a^
**Number of unprotected sex acts in past week**, median (IQR, range)		2 (0–3, 0–28)	0 (0-0, 0–10)	0 (0-0, 0–12)	<0.001^a^	<0.001^a^
**Reasons for inconsistent condom use in past 3 months** (multiple-response question), n/N§	Partner looked healthy	0/233	0/61	1/38	-	-
	Partners pays more without condom	2/233	14/61	13/38	-	-
	Forgot, had too much alcohol/drugs	1/233	2/61	1/38	-	-
	Embarrassed	0/233	0/61	1/38	-	-
	Fear for violence if using condom	2/233	0/61	0/38	-	-
	Child wish	6/233	0/61	0/38	-	-
	Trust the partner	143/233	8/61	1/38	-	-
	Partner refused	85/233	43/61	32/38	-	-
	Partner tested for HIV	18/233	1/61	0/38	-	-
Partner-level analysis						
**Total number of partners in past week**		259	456	632	<0.001^a^	<0.001^a^
**Number of sex acts per partner in past week**, median (IQR, range)		3 (2–4, 1–28)	2 (1–3, 1–14)	1 (1–2, 1–7)	<0.001^a^	<0.001^a^
**Total number of unprotected sex acts in past week**		603	125	98	<0.001^a^	<0.001^a^
**Number of unprotected sex acts per partner in past week**, median (IQR, range)		2 (0–3, 0–28)	0 (0-0, 0–7)	0 (0-0, 0–5)	<0.001^a^	<0.001^a^
**Proportion of partners having unprotected sex acts with sex worker in past week**, % (n/N)	0 unprotected sex acts	27.1% (70/258)	86.6% (395/456)	91.0% (574/631)		
	1 unprotected sex act	18.6% (48/258)	6.8% (31/456)	5.1% (32/631)		
	2–3 unprotected sex acts	35.7% (92/258)	4.4% (20/456)	3.3% (21/631		
	4 or more unprotected sex acts	18.6% (48/258)	2.2% (10/456)	0.6% (4/631)		
					<0.001^b^	<0.001^b^
**Proportion of partners having unprotected sex acts with sex worker in past week, by age-discordance**, % (n/N)¥	Partner 3 age groups older	0	20.0% (1/5)	9.1% (1/11)	-	-
	Partner 2 age groups older	57.1% (12/21)	14.3% (8/56)	6.4% (5/78)	-	-
	Partner 1 age group older	74.2% (89/120	12.4% (24/193)	9.5% (24/254)	-	-
	Partner, sex worker same age group	74.1% (80/108)	14.0% (23/164)	10.0% (25/250)	-	-
	Partner 1–2 age groups younger	75.0% (6/8)	14.7% (5/34)	6.3% (2/32)	-	-

¥ Age groups consist of <25 years; 25–34 years; 35–44 years; 45 years and older.

§ Information was obtained using a 3 month recall period in which female sex worker reported sex with boyfriend(s)/husbands (n = 290), regular clients (n = 266) and casual clients (n = 277).

a Poisson regression controlled for multiple measures on the same subject.

b Ordinal logistic regression controlled for multiple measures on the same subject.

At endline, 239 women with emotional partners reported sex in the past week with 259 emotional partners, leading to a total of 603 unprotected sex acts. Despite having a larger total number of regular clients (n = 456), and casual clients (n = 632), the corresponding total number of reported unprotected sex acts in the past week with these clients was 125 and 98, respectively. Age difference between the sexual partner and the sex worker was not associated with unprotected sex acts (table 2).

HIV incidence for all study participants was 2.6 infections per 100 person years (95% confidence interval [CI] = 1.4–4.9), and incidence of any STI (HIV, syphilis or *Trichomonas vaginalis*) was 8.7/100 years (95%CI = 6.2–12.3). Women with an emotional partner had a similar incidence of HIV to other women, but the hazard ratio (HR) of acquiring any STI was 2.2-fold that of other women (95%CI = 0.76–6.4; *P* = 0.15). At study end, 0.8% of women reporting consistent condom use with emotional partners had acquired HIV (1/134), while acquisition of HIV occurred in 2.6% of other women (*P* = 0.22). Pregnancy was 2.0 fold more common in women with an emotional partner, than other women (95%CI HR = 1.1–3.4; *P* = 0.021). Level of relationship control was not associated with biological outcomes.

### Reasons for non-use of condoms and perceived risk of HIV

Sixty one per cent of participants (143/233) attributed trust in their partner as the main reason for not using condoms with their emotional partner. This reason was seldom cited with regular or casual clients, where main barriers to condom use were partners' refusal (70.5% [43/61] of women with a regular client and 84.2% [32/38] with casual clients), and increased financial gain for unprotected sex (mentioned by 23.0% [14/61] and 34.2% [13/38] of women with regular and casual clients, respectively). Women who perceived themselves to be at moderate or high risk for HIV infection (n = 166), were asked to explain this in an open-ended question. The majority (77.7%; 129/166) reported this risk to be associated with clients' behaviour, and provided reasons illustrating this. For example, a 19-year old single Protestant woman who, in the past three months, always used condoms with clients but never with her boyfriend, perceived herself to be at great risk and said:

‘I have multiple sex partners. Even though I use condoms with them, the condoms can break, tear or even slip off, putting me at high risk.’

Similarly, a 21-year old, recently separated woman who, in the past three months always used condoms with clients but never with her boyfriend, recognized herself to be at high risk and reported:

‘Sex work in itself is risky, even with condom use because condoms can burst’.

Only 9 (5.4%) women mentioned HIV risk related to an emotional partner, with an additional 7 women reporting feeling at risk from both clients and emotional partners. A 31-year old married FSW who saw herself to be at moderate risk of becoming infected with HIV due to the behaviour of her husband provided the following reason:

‘Because I don't know how many people my husband has sex with, so even if I protect myself he might infect me because he doesn't like to use condoms.’

### Level of relationship control

More than a third of women (108/293) perceived themselves as having low control over their relationships with emotional partners, while only 10.6% of women (31/293) reported high relationship control (table 3). The median score on the relationship control scale was 28 (IQR = 20–34). Women with no or incomplete primary education were significantly more likely to have low control over their relationships compared to women with primary or higher education (*P*<0.001; data not shown). The level of relationship control was also associated with condom use in emotional partnerships. In a stepwise manner, inconsistent condom use in the past three months rose as relationship control decreased (*P*<0.001). Similarly, no women with high relationship control had been physically forced to have sex by their emotional partners. Conversely, this was reported by 3.9% (6/154) and 9.3% (10/98) of women who had medium or low control, respectively (*P* = 0.063).

**Table 3:** Association between level of control that FSWs perceive in their relationship with their emotional partners (n = 293), and sexual behaviour and sexual violence at the final visit of the 12-month cohort study (2006–2007).

Variable	Level of control in relationship with boyfriend(s)/husband¥			*P value* ¶
	High (score 37–48) n = 31	Medium (score 25–6) n = 154	Low (score 12–24) n = 108	
**Total number of sexual partners in past week**, median (IQR)	3 (1–6)	3 (1–5)	4 (1–6)	0.052§
**Inconsistent condom use with boyfriend(s)/husband in past 3 months**, % (n/N)	53.6% (15/28)	79.2% (118/149)	89.7% (96/107)	<0.001
**Refused sex in past week because boyfriend(s)/husband declined to use condom**, % (n/N)	22.6% (7/31)	10.4% (16/154)	13.9% (15/108)	0.17
**Ever physically forced by boyfriend(s)/husband to have sex**, % (n/N)	0/31	3.9% (6/154)	9.3% (10/98)	0.063

¶ Pearson X^2^ test for trend unless otherwise indicated.

§ Kruskal Wallis.

¥ 12 questions used, each scored from 1 (strongly agree) to 4 (strongly disagree). Total cumulative scores range from 12 (lowest relationship control) to 48 (highest relationship control).

### Experience of violence

Sexual and/or physical violence by an emotional partner was experienced by over half (55.0%; 202/367) of FSWs over the 12-month study period and was associated with a higher number of partners (*P* = 0.045) and inconsistent condom use (*P*<0.001; data not shown). Over a quarter (94/367) reported being slapped or having something thrown at them by any partner in the preceding 12 months, and 5.7% (21/367) had experienced this ‘many times’ (table 4). Nearly a quarter of participants had ever been physically forced to have sex by any partner when they did not want sex (79/367). Eleven participants (3.0%) reported being physically forced to have sex ‘many times’ in the preceding year, while 16.1% (59/367) had been physically forced to have sex once or a few times. In 43.0% (34/79) of women reporting to have been physically forced to have sex, this was by a stranger; in 38.0% (30/79) it was by someone she knew, and in 21.5% (17/79) it was by an emotional partner (data not shown).

**Table 4:** FSWs' experience of violence from current boyfriend or husband, or any other partners in the 12 months prior to cohort entry and during the 12 months cohort (2006–2007).

Variable assessing occurrence in past 12 months	Sub-category	Baseline, % (n/N)	After 12 months, % (n/N)
Physical violence			
**Pushed or shoved by partner**	**Yes, at least once**	**31.8% (127/399)**	**26.7% (98/367)**
	once	13.3% (53/399)	10.6% (39/367)
	a few times	13.3% (53/399)	8.4% (31/367)
	many times	5.3% (21/399)	7.6% (28/367)
**Slapped by partner or something thrown at her that could hurt**	**Yes, at least once**	**23.5% (94/400)**	**25.6% (94/367)**
	once	9.3% (37/400)	12.8% (47/367)
	a few times	10.3% (41/400)	7.1% (26/367)
	many times	4.0% (16/400)	5.7% (21/367)
**Hit with a fist or something else, kicked, or beaten up by partner**	**Yes, at least once**	**14.3% (57/400)**	**13.6% (50/367)**
	once	4.3% (17/400)	4.6% (17/367)
	a few times	7.0% (28/400)	4.9% (18/367)
	many times	3.0% (12/400)	4.1% (15/367)
Sexual violence			
**Physically forced to have sex with partner**	**Yes, at least once**	**22.3% (89/400)**	**19.1% (70/367)**
	once	11.3% (45/400)	12.0% (44/367)
	a few times	9.8% (39/400)	4.1% (15/367)
	many times	1.3% (5/400)	3.0% (11/367)
**Had sex with partner as was afraid of what he might do**	**Yes, at least once**	**17.8% (71/398)**	**31.3% (115/367)**
	once	7.3% (29/398)	13.9% (51/367)
	a few times	8.0% (32/398)	11.7% (43/367)
	many times	2.5% (10/398)	5.7% (21/367)
**Forced by partner to do something sexual which she found degrading or humiliating**	**Yes, at least once**	**10.8% (43/397)**	**12.0% (44/366)**
	once	5.8% (23/397)	7.4% (27/366)
	a few times	2.8% (11/397)	3.0% (11/366)
	many times	2.3% (9/397)	1.6% (6/366)

## Discussion

Sex workers' relations with their boyfriends or husbands were characterised by high-risk sexual behaviour, low levels of control over these relationships and frequent violence, each discussed further hereafter.

### Sexual behaviour and condom use

While number of unprotected sex acts [18], [19]and having multiple partners [20], [21] are critical factors influencing the transmission of HIV, other contributing factors include sexual networks, type of sex, HIV-status of partner, HIV disease stage, presence of STIs and circumcision [22], [23]. The hierarchy of these risk factors within an African sex work setting is not definitively known, but unprotected sex is of key importance. Over the course of our study, only 10–20% of women consistently used condoms with emotional partners, while more than 70% reported consistent use with their clients, similar to other studies in the region [8], [9], [24]. A study in Kenya, as in our research, found that the median number of unprotected sex acts was much greater for emotional partners than for clients, thus supporting the premise that unsafe sex between FSWs and their emotional partners may contribute more to HIV transmission than unsafe sex with clients [10]. Our participants reported a six-fold higher number of unprotected sex acts per week with emotional partners (with as many as 600 contacts), as compared to those with regular clients or casual clients.

There are further risks in FSWs' interactions with emotional partners, as these men often engage in other high-risk behaviours [7]. Almost half of the FSWs interviewed in a study in Pretoria, South Africa, reported that their emotional partners had concurrent partners [25]. High levels of concurrency were also found in Guinea and Benin, where 70% of emotional partners of FSWs noted that they were also clients of one or more sex workers [26]. Further, HIV prevalence among emotional partners of FSWs has been shown to be higher than that among FSW clients [27], [28]. In assessing HIV risk, these patterns suggest that sex work in sub-Saharan Africa cannot be viewed narrowly, or single dimensionally as sex worker and client interactions, but must specifically target the emotional partners of FSWs too.

### Relationship control

A third of women in our study had low control of their relationships with emotional partners, which was linked to higher numbers of partners, inconsistent condom use, and being physically forced to have sex by their emotional partners. A study with 15–24-year old women in South Africa concluded that women with low relationship control were twice as likely to use condoms inconsistently, and that those who had been forced into sex were nearly six times more likely not to use condoms consistently with that partner [29]. Similarly, a study among antenatal attendees in Soweto, South Africa found intimate partner violence and high levels of male control in a woman's current relationship were associated with her being HIV positive [16].

It is of concern that only 13.0% (38/293) of women in our study turned sex down because their intimate partners did not want to use condoms – this is either because the women did not perceive the sexual act to be sufficiently risky, or felt that they could not refuse sex. Studies have offered several reasons for sex worker reluctance to use condoms with their emotional partners, including a desire to distinguish between interactions with clients and those with emotional partners; the belief that a steady partner is HIV negative or ‘trustworthy’ (perhaps a more nuanced description of ‘trust’ recorded in our study); and the wish to avoid appearing suspicious in emotionally significant relationships, as would be implied by a request for condom use in a context where cultural norms of intimacy and trust suggest that one is protected [30]–[32]. Moreover, emotional partners and regular clients are also often regarded by FSWs as ‘clean’ and ‘safe’, in contrast to unknown casual clients, who may be viewed as ‘dirty’ and ‘unsafe’ [33], [34]. Although sex workers generally perceive themselves to be at very high risk of HIV, many consider this risk emanating from clients, rather than from emotional partners [35]. This ‘risk perception bias’ with intimate partners should be targeted in public health interventions relating to sex work [24].

### Experienced physical and sexual violence

Power within the relationship has a clear causal link with violence (or the threat thereof), which impacts on health [36]. Partner violence experienced by this cohort was high in comparison with women from the general population in Kenya. The Kenyan Demographic and Health Survey of 2003 found that 38.5% (1662/4312) of women ever experienced physical violence and 14.1% (606/4312) were ever subjected to sexual violence by an intimate partner – our study found close to double these figures. The latter is in line with other studies on FSWs in a variety of contexts [37]–[39].

### Working with men

Ward et al. point out that the health risks of sex work are two-fold – direct and indirect – and that services and programmes cannot be limited to the risks posed directly by clients [5]. Our results point to the urgent need for interventions targeting all males who are sexually associated with FSWs to take responsibility for protective sexual intercourse, reducing sexual entitlement, and promoting gender equality within relationships [40] – and particularly to focus on the emotional partners of sex workers.

### Study limitations

Study participants were enrolled through peer networks (non-random convenience sampling) in two divisions of Mombasa town and may therefore not fully represent the total sex worker population, although characteristics are similar to other studies in the region [8], [41], [42]. The study only enrolled HIV uninfected women and findings may not apply to women already infected with HIV. Information obtained on the sexual and other behaviours of emotional partners was through interview with the FSWs. Increased reliability of the data could be obtained with direct information from emotional partners and clients. It would be particularly pertinent to assess relationship control from the perspective of the partners. Further, the distinctions drawn between regular clients, casual clients and emotional partners are possibly an over-simplification, and possibly men move between these groups over time. Sexual risk behaviour in our study was assessed by describing condom use with each of the different types of partners, but information regarding the emotional partner's HIV status would more clearly determine HIV risk. At the same time, the study enquired about ‘sex acts’ and did not assess the type of sex involved. Some sex acts may have included masturbation or oral sex, where a condom may not be deemed necessary and HIV risk is low. Finally, repeated risk reduction counselling at clinic visits may have heightened social desirability bias in participants over time, and thus might account, in some part, for the reduction in reported inconsistent condom use.

In conclusion, frequent violence, low control and high-risk sexual behaviour in relationships with emotional partners highlight FSWs' vulnerability and high HIV risk, particularly through sexual interactions with their emotional partners. Sex worker risk in sub-Saharan Africa cannot be limited to sex worker and client interactions, but should explicitly include the emotional partners of sex workers. Programmes raising awareness of HIV risk within the sex work community should focus on all unprotected sexual intercourse – not on the type of sexual partner or emotional connection with a sex partner – and strategies for mediating that risk. At the same time, sex worker empowerment and structural interventions should include components that focus on power and control within intimate relationships, and negotiation strategies of protected sex with emotional partners.

## Supporting Information

Table S1 **Data supporting** **figure 1** **.** FSWs reporting inconsistent condom use in past 3 months.
(DOCX)
