# Supplementary material for: The Contribution of Emotional Partners to Sexual Risk Taking and Violence among Female Sex Workers in Mombasa, Kenya: A Cohort Study
Source: PLoS One. 2013 Aug 7;8(8):e68855. doi: 10.1371/journal.pone.0068855 (PMC3737234; doi:10.1371/journal.pone.0068855)
Supplement: Table S1 — Data supporting figure 1 . FSWs reporting inconsistent condom use in past 3 months. (DOCX) [file pone.0068855.s001.docx]

| **Variable** | **Sub-category** | **Month 0** | **Month 3** | **Month 6** | **Month 9** | **Month 12** | ***P**** |
| --- | --- | --- | --- | --- | --- | --- | --- |
| **Inconsistent condom use in past 3 months,** % (n/N) | With boyfriend(s)/ husband | 86.7% (248/286)) | 89.8% (274/305) | 82.3% (228/277) | 85.2% (236/277) | 80.3% (233/290) | 0.007 |
|  | With regular client | 38.8% (153/394) | 29.7% (105/354) | 24.6% (82/334) | 20.6% (65/316) | 22.9% (61/266) | <0.001 |
|  | With casual client | 32.5% (117/360 | 16.4% (54/329) | 13.9% (43/310) | 11.5% (34/297) | 13.7% (38/277) | <0.001 |

***** Generalized estimating equations (GEE) were used to assess changes in condom use over time, accounting for repeat measures
